# Drug-Drug Interactions in Transgender Patients Receiving Antiretroviral and Hormonal Therapy: A Systematic Review of Clinical Studies and Reports

**DOI:** 10.7759/cureus.81918

**Published:** 2025-04-08

**Authors:** Riya Yadav, Ekta Krishna, Bhoomika Shrivastava, Juhi M Singh, Madhusudan P Singh

**Affiliations:** 1 Pharmacology, All India Institute of Medical Sciences, Raipur, Raipur, IND; 2 Community and Family Medicine, All India Institute of Medical Sciences, Patna, Patna, IND; 3 Obstetrics and Gynecology, Jakir Hossain Medical College and Research Institute, Murshidabad, IND; 4 Pathology, K.D. (Kanti Devi) Medical College, Mathura, IND

**Keywords:** antiretroviral therapy, drug-drug interactions, highly active (haart), hormone replacement therapy, pharmacokinetics, transgender persons

## Abstract

Transgender individuals, whose gender identity differs from their assigned sex at birth, often undergo pharmacotherapy, including hormone therapy (HT), as part of their gender-affirming care. This pharmacotherapy, particularly when combined with antiretroviral therapy (ART) for transgender individuals living with HIV, raises concerns about drug-drug interactions (DDIs). These interactions can alter treatments' efficacy and lead to adverse health outcomes, making understanding their nature and extent critical. Despite the importance of identifying and managing DDIs in this population, limited comprehensive data are available.

This systematic review aims to collate and synthesize existing evidence from clinical studies and case reports on DDIs in transgender patients, with a specific focus on those receiving ART for HIV. A comprehensive literature search was conducted across multiple databases, including PubMed, Cochrane Library, and Scopus, following a predefined protocol registered with PROSPERO. Studies reporting DDIs in transgender patients undergoing any form of pharmacotherapy were included, and data extraction and quality assessment were performed independently by two reviewers.

The review found that while pharmacokinetic (PK) changes due to DDIs occur, they are often not clinically significant, suggesting that HT and ART can generally be co-administered safely in transgender individuals. However, specific interactions, such as those involving efavirenz-based ART, may require careful monitoring and individualized treatment adjustments. The findings highlight the need for tailored approaches in managing DDIs to optimize treatment efficacy and safety in transgender patients.

In conclusion, this review underscores the importance of further research into DDIs in transgender populations, particularly those on ART, to better inform clinical practice and improve patient care. Understanding these interactions is vital to ensuring optimal treatment outcomes and enhancing the quality of life for transgender individuals.

## Introduction and background

Transgender individuals, those whose gender identity differs from the sex they were assigned at birth, often undergo pharmacotherapy as part of their gender-affirming care [1]. This pharmacotherapy typically includes hormone therapy (HT), which may involve estrogen or testosterone administration, alongside other medications that may be prescribed for various health conditions [2]. As with any population undergoing multiple drug therapies, the risk of drug-drug interactions (DDIs) is a significant concern. These interactions can alter the efficacy of treatments and potentially lead to adverse health outcomes, making it crucial to understand the nature and extent of DDIs in transgender patients [3]. One of the most critical outcomes is reduced ART efficacy due to altered drug levels, which may result in virologic failure, increased risk of HIV transmission, and the development of drug resistance. On the other hand, interactions affecting hormonal therapy can lead to suboptimal feminization or masculinization, hormonal imbalance, and a potential worsening of gender dysphoria. Additionally, increased concentrations of estrogens or androgens due to DDIs may predispose individuals to thromboembolic events, liver dysfunction, or metabolic disturbances. These adverse outcomes underscore the importance of proactive DDI screening, personalized medication management, and interdisciplinary collaboration in the care of transgender patients on ART.

HIV disproportionately affects the transgender population, especially transgender women, who face higher risks due to factors such as discrimination, limited access to healthcare, and high rates of sex work, which increase vulnerability to HIV transmission compared to the general population [4,5]. ART is essential for managing HIV, but its concomitant use with HT presents unique challenges. Both estrogen and testosterone can influence the metabolism of ART drugs, potentially affecting their efficacy and safety. Conversely, some ART drugs may alter the metabolism of hormones, impacting the desired outcomes of gender-affirming therapies [6,7]. In addition to gender-affirming HT (GAHT), transgender individuals may also be prescribed medications such as warfarin for coexisting conditions, which can further complicate therapy due to potential interactions with ART. Notably, this review also includes case reports evaluating interactions between ART and warfarin, highlighting the broader clinical implications of DDIs beyond hormonal agents. Recognizing and managing these interactions is essential to optimizing therapeutic outcomes and minimizing adverse effects in this population.

DDIs in the transgender population are an important clinical concern. Yet, they remain understudied and underreported, possibly due to challenges such as small sample sizes, stigma, and the complex interplay of medications used in this population [8]. Despite the importance of identifying and managing DDIs in this population, there is a paucity of comprehensive data synthesizing the scope and clinical significance of DDIs among transgender patients [9].

Given the complex pharmacokinetics (PK) and pharmacodynamics associated with these therapies, understanding DDIs in transgender individuals, particularly those on ART, is vital [10]. Unawareness of these interactions can lead to suboptimal treatment outcomes, increased side effects, and diminished quality of life [11,12]. Therefore, there is an urgent need for a comprehensive review of DDIs specific to this population to inform clinical practice and improve patient care.

This systematic review aims to collate and synthesize available evidence from clinical studies and case reports regarding DDIs in transgender individuals, with a particular emphasis on those receiving ART for HIV. The primary research question guiding this review is: What are the clinically significant DDIs reported in transgender patients on ART, and how do these interactions impact treatment outcomes and patient safety? We hypothesize that certain combinations of ART with GAHT and other commonly prescribed medications (e.g., warfarin) pose a measurable risk of reduced therapeutic efficacy or increased adverse effects. By addressing this question, the review seeks to equip healthcare professionals with practical insights for identifying, preventing, and managing DDIs in transgender care.

## Review

Methodology

This systematic review followed a predefined protocol registered with PROSPERO (CRD42024555356). The Preferred Reporting Items for Systematic Reviews and Meta-Analyses (PRISMA) guidelines were used to conduct the review [13].

Search Strategy

A comprehensive literature search was performed in PubMed, Cochrane Library, ClinicalTrials.gov, Web of Science, and Scopus databases using a combination of relevant keywords and subject headings related to "transgender", "drug-drug interactions", "case reports", "case series", "observational studies", and "randomized controlled trials". The search covered studies published from inception to the current date. No language or date restrictions were applied.

Eligibility Criteria

Studies reporting DDIs in transgender patients receiving any form of pharmacotherapy, including HT and antiretroviral therapy (ART), were included. Case reports, case series, observational studies (cohort and case-control studies), and RCTs were considered for inclusion. For the purposes of this review, a DDI was defined as any documented alteration in the PK (e.g., absorption, distribution, metabolism, or excretion) or pharmacodynamics of a drug when co-administered with another agent in transgender individuals receiving ART. Both studies reporting PK evidence, such as changes in drug concentrations or enzyme activity, and those documenting clinical outcomes, including therapeutic failure or adverse events attributable to suspected interactions, were included. This inclusive definition was adopted to capture the broad spectrum of clinically relevant interactions, whether mechanistically or outcome-driven. Studies not reporting primary data on DDIs or involving non-transgender populations were excluded. Additionally, reviews, editorials, and opinion pieces without primary data were excluded.

Study Selection and Data Extraction

Two reviewers independently screened the titles and abstracts of retrieved studies against the eligibility criteria. Disagreements were resolved through discussion with a third reviewer. Full texts of potentially eligible studies were obtained and evaluated for final inclusion. Data were extracted using a standardized form designed to capture relevant information, including study design, population characteristics, types of pharmacotherapy, nature of DDIs, clinical outcomes, and effect measures. Two reviewers independently extracted data, and any discrepancies were resolved by consensus.

Quality Assessment

The quality of included studies was assessed using established, study design-specific tools. For randomized controlled trials (RCTs), the Cochrane Risk of Bias Tool was used, which evaluates domains such as random sequence generation, allocation concealment, blinding, incomplete outcome data, and selective reporting [14]. For observational studies, the Newcastle-Ottawa Scale was applied [15], assessing quality based on selection of study groups, comparability of groups, and ascertainment of exposure or outcomes. For case reports and case series, we employed the Case Report (CARE) Quality Assessment Tool [16], which considers criteria such as clarity of clinical information, diagnostic methods, therapeutic interventions, and outcomes. Two reviewers independently performed all quality assessments, and any discrepancies were resolved through discussion or, when necessary, by consulting a third reviewer. Detailed scoring for each study is presented in the corresponding tables.

Data Synthesis and Analysis

A narrative synthesis of the findings was conducted, summarizing the types and clinical relevance of DDIs reported in the included studies. A meta-analysis was performed to pool quantitative data using a random-effects model if feasible and appropriate. Heterogeneity across studies was assessed using the I² statistic. Subgroup analyses were conducted based on the type of pharmacotherapy, the nature of DDIs, and demographic variables (e.g., age, gender identity).

Results

Study Results, Study Characteristics, and Baseline Demographics

**Figure 1:**
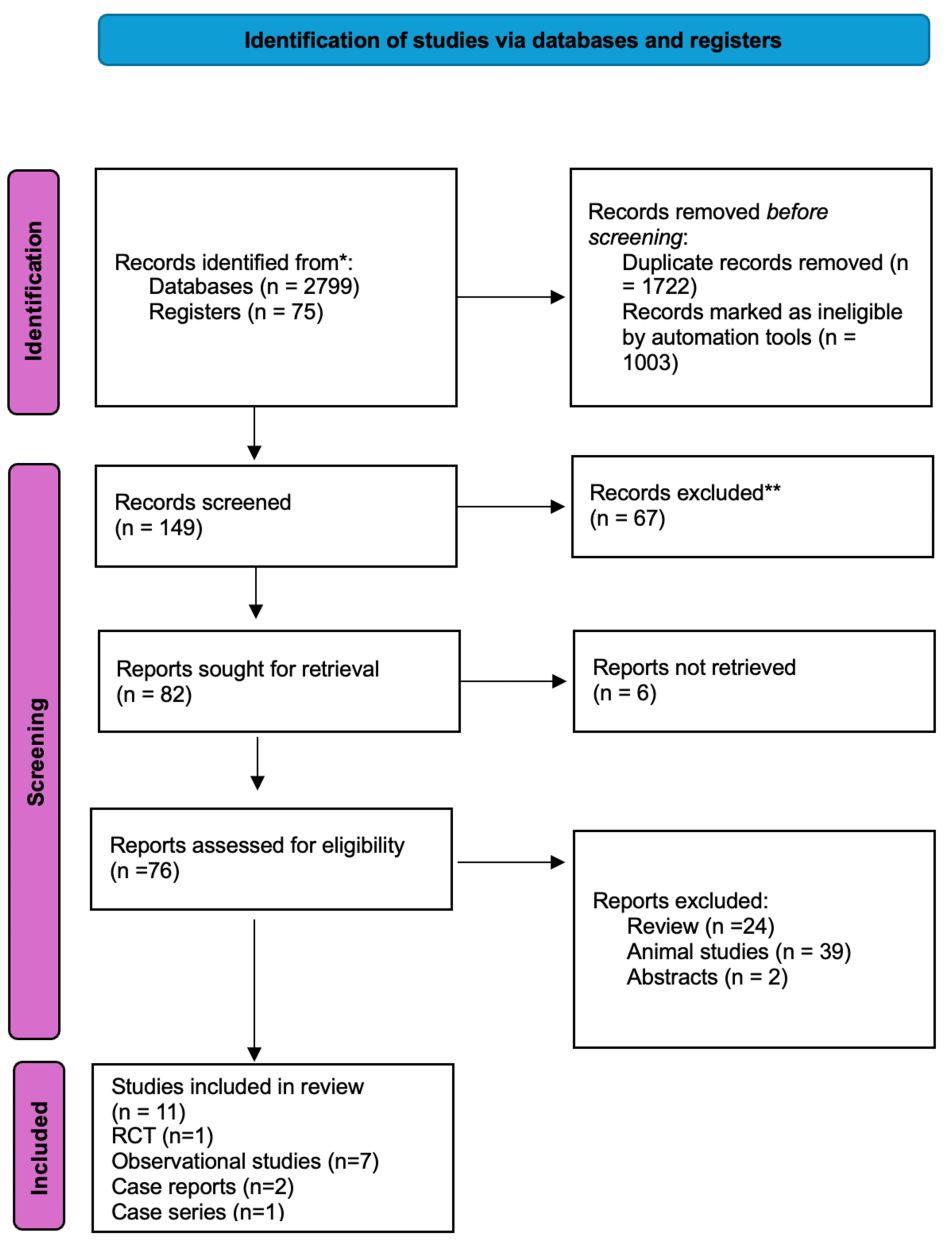
PRISMA flowchart PRISMA: Preferred Reporting Items for Systematic Reviews and Meta-Analyses, RCT: randomized controlled trials

A PRISMA flow chart (Figure 1) outlines the study selection process, starting with an initial pool of 2874 studies. The final analysis included one RCT, seven observational studies, two case reports, and one case series, encompassing a total of 1701 patients: 1690 from observational studies, six from the RCT, three from the case series, and two from the case reports.

Randomized Controlled Trials

The RCT by Lam et al. [17] assessed the PK interactions between doravirine, tenofovir, estradiol, and testosterone in transgender women receiving feminizing HT (FHT) and ART for HIV. The study found minor changes in drug exposure: doravirine decreased by 3.3%, tenofovir increased by 16.7%, and estradiol increased by 10.2%, but none were considered clinically significant (Table 1).

**Table 1:** Summary of RCTs and observational studies RCTs: randomized controlled trials, HIV: human immunodeficiency virus, No.: number, PK: pharmacokinetics, AUC: area under the curve, PrEP: pre-exposure prophylaxis, ART: antiretroviral therapy, d: per day, DBS: dried blood spots, PBMC: peripheral blood mononuclear cells, CrCl: creatinine clearance, TG: transgender, CG: cisgender, TGF: transgender female, TGM: transgender male, CGM: cisgender male, CGF: cisgender female, E2: estradiol, FHT: feminizing hormone therapy, FTC: emtricitabine, TFV: tenofovir, TDF: tenofovir disoproxil fumarate, TFV-DP: tenofovir diphosphate, FTC-TP: emtricitabine triphosphate

Author/year	Study design	No. of patients	Mean age (in years)	TGM or TGF	Disease condition/indication	Pharmacotherapy	DDIs	Results	Conclusion
Lam et al., 2023 [17]	RCT	Enrolled: 8, completed: 6	25.5 years	TGF: 8	HIV feminization of TG	Doravirine, TFV, lamivudine, E2, and spironolactone	Doravirine and TFV with E2 and spironolactone	Doravirine exposure was reduced by approximately 3.3%, while TFV exposure increased by about 16.7%. E2 exposure also increased by approximately 10.2%. There was no significant change in total testosterone levels.	There were no clinically meaningful changes in the PK of any of the drugs.
Armstrong et al., 2023 [18]	Observational study	1495	35	TGF: 1495	HIV feminization of TG	ART therapy, oral E2 valerate, and cyproterone	ART and E2	A total of 86 TGF with HIV were receiving ART. There was no significant difference in serum E2 levels between TGF with and without HIV. Serum testosterone levels were also similar across both groups.	TGF with HIV are prescribed FHT less frequently. However, there is no significant variance in serum E2 or testosterone levels. Additionally, there is minimal interaction between FHT and ART.
Cirrincione et al., 2020 [19]	Observational study	16	32	TGF: 16	PrEP of HIV, feminization of TG	TFV disoproxil fumarate, FTC, 17 beta-E2, and spironolactone	TFV and FTC with FHT	Lower plasma concentrations of TFV and FTC were observed compared to controls, while intracellular concentrations were higher than those in the control group.	Differences in PrEP PK are important and crucial for assessing its efficacy in TGF.
Cattani et al., 2023 [20]	Observational study	19	26	TGF: 19	PrEP of HIV, feminization of TG	ART therapy, E2, and spironolactone	ART therapy with E2 and spironolactone	There were no differences in E2 AUC and trough concentrations. However, the AUCs for spironolactone and canrenone were lower.	The PK of E2 remained unaffected by concurrent use of PrEP. Although minor discrepancies were noted, they are not clinically significant and should not discourage the simultaneous administration of E2-based FHT and PrEP.
Hiransuthikul et al., 2021 [21]	Observational study	20	25.5	TGF: 20	HIV feminization of TG	ART therapy E2 valerate 2 mg/d and cyproterone acetate 25 mg/d	TFV, FTC, and efavirenz with E2	The PK parameters of E2 are reduced in the presence of TFV, FTC, and efavirenz.	The PK parameters of TFV and efavirenz were diminished with FHT.
Shieh et al., 2019 [22]	Observational study	16	TGF: 29, CGM: 46	TGF: 8, CGM: 8	PrEP of HIV, feminization of TG	TFV, TDF/FTC, FHT	TFV, FTC with E2	Similar plasma TFV exposures were observed between TGM and women. However, higher FTC plasma exposures were found in TGM.	Plasma TFV exposures are similar between TGM and women, with higher FTC plasma exposures observed in TGM compared to TGF.
Grant et al., 2021 [23]	Observational study	74	30	TGM:TGF: 23:2, CGM:CGF: 15:17	PrEP of HIV, gender-affirming therapy	Oral FTC/TDF. Testosterone or E2 therapy	FTC, TFV with testosterone or E2	Testosterone and E2 levels remained unchanged after 4 weeks of PrEP in both TGF and TGM. TFV-DP concentrations were similar across TGF, TGM, and CGM. However, lower TFV-DP levels were observed in TGM compared to CGF.	After four weeks of FTC/TDF PrEP, similar levels of TFV-DP were observed in CGM, TGM, and TGF. Serum hormone levels were unaffected by PrEP use.
Yagger et al., 2022 [24]	Observational study	50	TGF: 19.9, TGM: 20.3	TGF: 26, TGM: 24	PrEP of HIV, gender-affirming therapy	FTC/TDF, E2–spironolactone or testosterone	TFV, FTC with E2 or testosterone	Plasma TFV exposures were similar between TGM and TGF. FTC plasma exposures were 21% higher in TGM than in TGF. TFV-DP levels in PBMC and DBS, as well as FTC-TP in DBS, were similar between TGM and TGF after adjusting for CrCl. However, FTC-TP in PBMC was 46% higher in TGM than in TGF. All PK exposures were within expected ranges based on historical studies.	All PK exposures were within the anticipated ranges based on historical studies. TM exhibited higher FTC exposures than TW. Plasma and intracellular exposures for both drugs were consistent with historical studies. High PrEP efficacy is expected in adolescent and young adult TG individuals.

Observational Studies 

Armstrong et al. [18] found no significant differences in serum estradiol or testosterone levels between transgender women with and without HIV, suggesting minimal interaction between FHT and ART. Cirrincione et al. [19] observed lower plasma concentrations of tenofovir and emtricitabine in transgender women on pre-exposure prophylaxis (PrEP) with FHT, though intracellular concentrations were higher, potentially impacting PrEP efficacy. Cattani et al. [20] reported no significant differences in estradiol exposure when combined with tenofovir/emtricitabine PrEP, though minor discrepancies in spironolactone exposure were noted. Hiransuthikul et al. [21] found reduced estradiol PK when co-administered with tenofovir, emtricitabine, and efavirenz in HIV-positive transgender women on FHT (Table 1).

Case Reports and Series

Liedtke et al. [25] described a case where a transgender woman required a 45% increase in weekly warfarin doses after starting a ritonavir-boosted ART, necessitating enhanced INR monitoring (Table 2). Leinung et al. [26,27] reported low serum estradiol levels in two transgender women on efavirenz-based ART, suggesting the need for hormone level monitoring and potential dose adjustments (Table 2).

**Table 2:** Summary of case reports and case series HIV: human immunodeficiency virus, DVT: deep vein thrombosis, TGF: transgender female, TGM: transgender male, ART: antiretroviral therapy, TRIO regimen: a combination regimen consisting of ritonavir-boosted darunavir, etravirine, and raltegravir, INR: international normalized ratio, TB: tuberculosis, TG: transgender, ATT: anti-tuberculosis therapy, FHT: feminizing hormone therapy, MPA: medroxyprogesterone acetate, β: beta (referring to 17-β Estradiol, a form of the estrogen hormone)

Author/year	Study design	No. of patients	Mean age (in years)	TGM or TGF	Disease condition/indication	Pharmacotherapy	DDIs	Results	Conclusion
Liedtke et al., 2012 [25]	Case report	1	50	TGF: 1	HIV, recurrent DVT	Antiretroviral TRIO regimen (ritonavir-boosted darunavir, etravirine, and raltegravir), warfarin	ART with warfarin	The weekly warfarin doses were increased by 45% following the TRIO regimen, and stable anticoagulation was maintained.	The concurrent use of the ART TRIO regimen with warfarin requires an increased dose of warfarin. Enhanced monitoring of the INR is necessary.
Suchak et al. 2024 [26]	Case report	1	30	TGF: 1	HIV, syphilis, TB, feminization of TG	ART (bictegravir/tenofovir alafenamide/emtricitabine, later dolutegravir), benzathine penicillin G ATT, ethinyl oestradiol, norelgestromin	ATT, ART, and FHT	I managed the rifampicin drug interaction by optimizing the HIV treatment and adjusted the estrogen dose accordingly.	The interaction between rifampicin and bictegravir required a switch in ART to dolutegravir. Estradiol levels were monitored and adjusted to maintain the therapeutic range.
Leinung et al., 2019 [27]	Case series	3	Patient 1: 29, patient 2: 56, patient 3: 52	TGF: 3	HIV, feminization of TG	Patient 1: efavirenz, tenofovir disoproxil fumarate, emtricitabine, estradiol, MPA. Patient 2: estradiol, efavirenz, tenofovir disoproxil fumarate, emtricitabine. Patient 3: estradiol patch, efavirenz, tenofovir disoproxil fumarate, emtricitabine	Efavirenz and estradiol	Patients 1 and 2 exhibited low serum 17-β estradiol levels.	Efavirenz affects estradiol metabolism. Therefore, it is important to tailor therapy for transgender women on efavirenz-based ART by monitoring hormone levels.

Drug-Drug Interactions

PK interactions were the most commonly reported, particularly with non-nucleoside reverse transcriptase inhibitors (NNRTIs) like efavirenz, which reduced serum estradiol levels. However, most studies show that FHT does not significantly affect the levels of antiretroviral drugs [27]. Pharmacodynamic interactions have also been observed, such as the increased need for warfarin dosing with ritonavir-boosted ART [25].

Clinical Significance of Interactions

While some studies reported no clinically meaningful changes in drug or hormone levels, others highlighted significant interactions, such as reduced estradiol levels with efavirenz-based ART and altered warfarin dosing with ritonavir-boosted ART [18-27]. Although the impact on clinical outcomes was not directly assessed, these interactions suggest the potential for suboptimal treatment responses if not properly managed.

Mechanisms of Interactions

Potential mechanisms for the observed DDIs include enzyme induction or inhibition by ART, particularly NNRTIs and ritonavir, affecting the metabolism of estradiol. Conversely, FHT generally did not appear to significantly alter the metabolism of antiretroviral or PrEP drugs [18-27].

Quality Assessment

All the studies included in this systematic review adhered to the relevant reporting guidelines, ensuring standardized and consistent presentation of case details. A detailed quality assessment can be found in Tables 3-4, which thoroughly evaluates the methodological quality of the included case reports and observational studies in this analysis.

**Table 3:** Quality assessment of observational studies using Newcastle Ottawa scale * one score, * two scores, *** three scores

S. No.	Study	Selection	Comparability	Outcome
1	Armstrong et al. 2023 [18]	***	*	*
2	Cirrinicione et al., 2020 [19]	***	*	**
3	Cattani et al., 2023 [20]	**	*	**
4	Hiransuthikul et al., 2021 [21]	**	*	***
5	Sheih et al., 2019 [22]	***	*	***
6	Grant et al., 2021 [23]	***	*	***
7	Yagger et al., 2022 [24]	***	*	***

**Table 4:** Reporting quality assessment of included case series/case reports using CARE quality assessment tool CARE: Case Report

Studies	Keywords	Abstract	Introduction	Patient information	Clinical findings	Timeline	Diagnostic intervention	Therapeutic intervention	Follow-up and outcomes	Discussion	Patient perspective	Informed consent
Liedtke et al., 2012 [25]	NO	YES	YES	YES	YES	YES	YES	YES	YES	YES	NO	NO
Suchak et al., 2024 [26]	NO	YES	YES	YES	YES	YES	YES	YES	YES	YES	NO	NO
Leinung et al., 2019 [27]	YES	YES	YES	YES	YES	NO	YES	YES	YES	YES	NO	NO

Discussion

This systematic review aimed to synthesize existing evidence on DDIs in transgender patients, focusing on those undergoing HT and ART. Our findings indicate that while certain PK changes occur due to DDIs, these changes are often not clinically significant, suggesting that HT and ART can generally be co-administered safely in transgender individuals. However, some exceptions and specific interactions warrant careful monitoring and individualized treatment adjustments.

Impact of Hormone Therapy on Antiretroviral Therapy

The RCT by Lam et al. [17] showed minimal PK changes when doravirine and tenofovir were co-administered with estradiol and spironolactone, consistent with other studies that found most ART regimens can be safely combined with HT without major dose adjustments [23]. However, Hiransuthikul et al. reported reduced estradiol PK with efavirenz-based ART, although this did not necessitate changes in hormone dosing [21].

Antiretroviral Therapy and Feminizing Hormone Therapy in Transgender Women With HIV

Armstrong et al. found no significant differences in serum estradiol levels between transgender women with and without HIV, suggesting minimal interaction between ART and FHT in this population. [18] This observation is crucial given that transgender women are at a higher risk for HIV and often require ART alongside FHT. The study’s findings are consistent with earlier reports that ART does not significantly impact hormone levels, thus supporting the concurrent use of these therapies [28-30].

However, some studies highlighted specific interactions that require attention. Cirrincione et al. reported lower plasma concentrations of tenofovir and emtricitabine but higher intracellular concentrations when co-administered with FHT [19]. This indicates a complex interaction where intracellular efficacy might be maintained despite lower plasma levels, suggesting that plasma monitoring alone might not suffice for assessing the efficacy of PrEP in transgender women [31,32].

Pre-exposure Prophylaxis and Hormone Therapy

The PK of estradiol remained unaffected by concurrent PrEP use, as Cattani et al. showed [20]. This supports the simultaneous administration of estradiol-based FHT and PrEP without compromising the efficacy of either therapy [33]. Additionally, studies by Shieh et al. [22] and Grant et al. [23] found that hormone levels remained stable with PrEP use, further affirming the safety of these combined regimens [22,23]. These results are in line with the conclusions from Yagger et al., who reported consistent PK exposures for both tenofovir and emtricitabine within expected ranges, ensuring high PrEP efficacy in transgender individuals [24].

Specific Drug Interactions

Several case reports and series provided insights into specific drug interactions. Liedtke et al. highlighted the need for increased warfarin doses when co-administered with an ART, emphasizing the importance of monitoring and dose adjustments in such scenarios [25]. Similarly, Suchak et al. demonstrated the significant interaction between rifampicin and bictegravir, necessitating a change to dolutegravir and carefully monitoring estradiol levels to manage the interaction [26].​​​​​​​

Tailoring Therapy for Transgender Individuals

The case series by Leinung et al. suggested that efavirenz affects the metabolism of oral estradiol, resulting in low serum estradiol levels [27]. This finding underscores the need for individualized hormone level monitoring to tailor therapy for transgender women receiving efavirenz-based ART [32]. These tailored approaches are essential for optimizing treatment efficacy and minimizing adverse effects in transgender patients [18].​​​​​​​

Comparison With Existing Literature

Our review corroborates the findings of previous systematic reviews and meta-analyses, which have generally concluded that most ART and HT regimens can be co-administered safely in transgender individuals. For instance, a review by Radix et al. noted that while some ART drugs might interact with hormone therapies, these interactions are typically manageable with appropriate monitoring and dose adjustments [10]. Our findings extend this knowledge by providing detailed insights into specific drug interactions and their clinical implications.

Moreover, our review highlights the importance of considering both plasma and intracellular drug concentrations when assessing the efficacy of PrEP in transgender individuals, as emphasized by Cirrincione et al. [19]. This dual monitoring approach ensures that PrEP efficacy is not compromised despite potential PK alterations in plasma levels [3,22,31].

Clinical Implications and Recommendations

The findings of this systematic review have important clinical implications, particularly for clinicians managing transgender individuals receiving GAHT and ART or PrEP. Awareness of potential DDIs is essential to optimize therapeutic outcomes, maintain viral suppression, and ensure the desired effects of HT. Regularly monitoring hormone levels and ART drug concentrations is critical to detecting and managing interactions early. This is especially important in combination with higher interaction risk, such as warfarin with ART or efavirenz with estradiol. Both plasma and intracellular concentrations (e.g., tenofovir diphosphate) should be considered when evaluating PrEP efficacy in transgender individuals. The reviewed evidence indicates that although some PK interactions between ART, PrEP, and GAHT exist, these are generally manageable with appropriate clinical vigilance. Based on this synthesis, the following practical recommendations are proposed:

Routine monitoring and dose adjustment: Clinicians should routinely assess hormone and ART levels, particularly when initiating or modifying regimens. Where possible, use therapeutic drug monitoring to guide dose adjustments, especially with drugs like warfarin and efavirenz.

Use of non-oral estradiol formulations: In cases of significant interaction between oral estradiol and ART, consider switching to transdermal or injectable estradiol formulations, which may bypass hepatic first-pass metabolism and reduce interaction potential.

Managing anticoagulation: Patients receiving warfarin should undergo close INR monitoring when initiating, discontinuing, or changing ART regimens. Dose titration may be necessary to maintain therapeutic anticoagulation.

Minimize polypharmacy risks: In low-resource settings, where diagnostic and therapeutic monitoring may be limited, minimizing unnecessary medications and simplifying ART or GAHT regimens can reduce DDI risks.

Education and counseling: Patient education regarding adherence, potential side effects, and signs of hormonal imbalance or ART failure is essential. Empowering patients with this knowledge can help bridge resource gaps and ensure timely reporting of complications.

Adaptation in low-resource settings: Where laboratory monitoring is limited, clinicians should rely on clinical signs and symptoms to guide therapy. Standardized symptom checklists, fixed-dose ART combinations, and less interaction-prone hormonal regimens may be practical alternatives.

PrEP considerations: Emerging evidence suggests that PrEP efficacy is maintained in transgender individuals on GAHT. However, clinicians should remain vigilant, particularly with long-acting formulations, and consider pharmacologic studies when available to guide dosing.

Integrative approach to care: Incorporating up-to-date clinical guidelines, pharmacogenetic data, and psychosocial support can further enhance patient care by tailoring treatments to individual metabolic profiles and addressing social determinants of health.

Limitations and Future Research

While this review provides comprehensive insights into DDIs in transgender patients, it is not without limitations. The heterogeneity of study designs and varying methodologies across studies may impact the generalizability of the findings. Additionally, the limited number of RCTs highlights the need for more robust clinical studies to confirm these observations.

Future research should focus on large-scale, multicenter RCTs to further elucidate the PK and clinical implications of DDIs in transgender patients. Studies should also explore the long-term effects of these interactions on health outcomes to provide more definitive guidance for clinicians.

## Conclusions

This systematic review highlights that while certain PK changes due to DDIs occur in transgender patients undergoing HT and ART, most are not clinically significant. However, specific interactions, such as those involving warfarin or efavirenz, require vigilant monitoring and individualized treatment adjustments. Regular monitoring of hormone and ART drug levels is advised in transgender patients on combined therapy to ensure optimal therapeutic efficacy and safety. Emphasizing tailored therapy and dose adjustments in response to DDIs is critical, for instance, considering non-oral routes of estradiol administration when interacting with ART agents. Moreover, developing comprehensive treatment regimens should carefully consider long-term effects and comorbidities, including impacts on bone mineral density, cardiovascular health, and other systemic issues. These findings underscore the need for an integrative, patient-centered approach and support the importance of ongoing research to provide clearer, evidence-based guidance for clinicians managing DDIs in transgender individuals.
